# Lifestyle Intervention on Metabolic Syndrome and its Impact on Quality of Life: A Randomized Controlled Trial

**DOI:** 10.5935/abc.20160186

**Published:** 2017-01

**Authors:** Patrícia Pozas Saboya, Luiz Carlos Bodanese, Paulo Roberto Zimmermann, Andreia da Silva Gustavo, Fabricio Edler Macagnan, Ana Pandolfo Feoli, Margareth da Silva Oliveira

**Affiliations:** 1 Universidade Federal de Ciências da Saúde de Porto Alegre - UFCSPA; RS - Brasil; 2 Pontifícia Universidade Católica do Rio Grande do Sul (PUCRS), RS - Brasil

**Keywords:** Metabolic Syndrome, Life Style, Quality of Life, Cardiovascular Diseases, Prevention, Risk Factors

## Abstract

**Background:**

Lifestyle intervention programs can reduce the prevalence of metabolic syndrome (MetS) and, therefore, reduce the risk for cardiac disease, one of the main public health problems nowadays.

**Objective:**

The aim of this study was to compare the effects of three types of approach for lifestyle change programs in the reduction of metabolic parameters, and to identify its impact on the quality of life (QOL) of individuals with MetS.

**Methods:**

A randomized controlled trial included 72 individuals with MetS aged 30-59 years. Individuals were randomized into three groups of multidisciplinary intervention [Standard Intervention (SI) - control group; Group Intervention (GI); and Individual Intervention (II)] during 12 weeks. The primary outcome was change in the metabolic parameters, and secondarily, the improvement in QOL measures at three moments: baseline, 3 and 9 months.

**Results:**

Group and individual interventions resulted in a significant reduction in body mass index, waist circumference, systolic blood pressure at 3 months and the improvement of QOL, although it was significantly associated with the physical functioning domain. However, these changes did not remain 6 months after the end of intervention. Depression and anxiety were significantly associated with worse QOL, although they showed no effect on the response to intervention.

**Conclusion:**

Multidisciplinary intervention, especially in a group, might be an effective and economically feasible strategy in the control of metabolic parameters of MetS and improvement of QOL compared to SI, even in a dose-effect relationship.

## Introduction

Metabolic syndrome (MetS), considered a complex set of cardiovascular risk factors related to abdominal fat and resistance to insulin, has been increasing progressively and is strongly associated with high cardiovascular morbimortality,^1,2^ with estimated prevalence around 23.7%, according to Adult Treatment Panel III criteria.^3^ The main recommendations for MetS prevention and treatment are the change in lifestyle through a multifactor approach based on education, regular physical exercise and a healthy diet, as well as pharmacological strategies.^1^

Studies show that programs of lifestyle change that include nutritional education and supervised physical exercise were efficient to achieve the proposed goals for the treatment of MetS.^4,5^ However, few studies use this multifactor approach in their interventions, including all main aspects in the intervention.^6-10^

Furthermore, an increasing number of studies support the idea that MetS is significantly associated with impaired quality of life (QOL),^11-13^ and that this association can be predictive of mortality.^14^ Otherwise, few intervention studies confirm the association between MetS and QOL, showing improvement in the MetS components, followed by better QOL scores after lifestyle change intervention,^7-10,15-17^ in up to 24 months of follow-up.^7^

Moreover, studies also show association between depression, anxiety and MetS, although they are not conclusive. While some studies demonstrate the association between MetS and depression,^18-21^ others reveal only association between MetS and anxiety.^22,23^ For this reason, the analysis of the prevalence of these clinical situations was carried out in this study in order to identify whether there is some influence of these variables in the recovery or the improvement process of the metabolic condition.

The study of prevention and treatment strategies, as well as the relationship between MetS and QOL, due to its relevance, complexity and treatment possibility, have been receiving little attention in medical literature. Thus, the aim of this study is to test three different programs with a multidisciplinary approach for lifestyle change in the reduction of metabolic parameters and QOL improvement in the population of a rapidly developing country.

## Methods

### Participants

Randomized controlled trial was conducted at the *Centro de Reabilitação do Hospital São Lucas da Pontifícia Universidade Católica do Rio Grande do Sul* (HSL-PUCRS), a general university hospital in Southern Brazil. The trial was registered in clinical trial registry Brazil, ReBEC, number RBR9wz5fc.

Inclusion criteria: waist circumference (WC) measure > 88 cm for females and > 102 cm for males, followed by at least two criteria: a) systolic blood pressure (SBP) ≥ 130 mmHg, diastolic blood pressure (DBP) ≥ 85 mmHg; b) triglycerides (TGL): ≥ 150 mg/dL; c) high-density lipoprotein cholesterol (HDL-C): < 40 mg/dL for males and < 50 mg/dL for females;^1^ and d) fasting glucose (FG): ≥ 100 mg/dL.^2^

Exclusion criteria: a) absolute contraindication for physical activity due to musculoskeletal, neurological, vascular, lung and cardiac problems; b) pregnancy; c) diagnosis of severe psychiatric disorders, significant cognitive impairment, assessed by the Mini Mental State Examination (scores under 24 as a cutoff point); d) unavailability to participate in the program.

### Procedures

Individuals recruited by media advertising in newspapers, radio and websites participated in a screening meeting when they were told about the objectives, inclusion and exclusion criteria of the study. After identifying the participants who were able to join the study, they were consecutively randomized into the three kinds of intervention for lifestyle change, by simple randomization 1:1:1. This procedure occurred successively in four waves till the sample size was reached.

After randomization, each individual received the information regarding the procedures involved in the study, specific for each program, and signed the written informed consent previously approved by the Ethics Committee in Research of PUCRS, under number 10/05153. Initial interviews were scheduled, as well as the following appointments, according to the intervention program drawn. All interviews and interventions were previously confirmed by telephone and performed by the researchers, who were submitted to quality standard training for data collection and intervention procedures.

#### Standard intervention

The standard intervention (SI), considered in this study as the control group, was the non-pharmacological intervention recommended by the main guidelines for the clinical management of MetS. The volunteers in this group had two consultations: at baseline and 3 months. Consultations were carried out individually by the nursing staff: the first one for standard guidance on exercising, diet and self-care, according to the guidelines. The diet program is based on the healthy diet model of the Brazilian Ministry of Health^24^ and the self-care program, focused on the administration of the medications in use and general health care. The second consultation approached the facility and difficulty to follow recommendations for changing eating habits and regular exercising.

#### Intervention group

The group intervention (GI) worked the change in lifestyle through the discussion of pre-defined themes of health education, focused on the main cardiovascular risk factors considered changeable which are associated with MetS, as well as motivation for changing behavior, based on the transtheoretical model of change.^25^ The GI appointments occurred weekly during 1 hour and 45 minutes, coordinated by a psychologist, a nurse, a physical therapist and a nutritionist. During the first 45 minutes, volunteers discussed a health topic proposed by the team. Soon after that, they discussed and tested strategies for changing eating habits and regular exercising, which could be included in the volunteers' routine, according to the group's motivation. The groups were composed of 10 to 12 individuals.

#### Individual intervention

The volunteers in the individual intervention (II) group participated in weekly individual appointments with the psychology and nutrition teams, and exercised regularly with the physical therapy team.

Nutritional intervention: based on the needs of each participant according to the aspects that should be changed, respecting intrinsic and extrinsic conditions necessary for the changing process of eating habits. During the weekly appointments, body weight was measured and adhesion to the diet program was assessed through a brief 24-hour recall. In addition, possible difficulties in the adhesion to the strategies and goals agreed in the previous consultation were constantly recorded and monitored. MetS-related themes were developed based on a pre-defined program and addressed individually, aiming to improve the understanding and adhesion to the strategies for changing eating habits.

Psychological intervention: based on the transtheoretical model of change,^25^ adapted for individual model, which worked on the different stages of change based on a structured program, with pre-defined objectives, as well as the specific change processes. Materials such as flyers were used and filled out by the volunteer.

Physical intervention: composed of 36 sessions on the treadmill for 60 continuous minutes each. They occurred three times a week, and the intensity was adjusted according to the recommended heart rate (HR) for each individual. The training range remained between 75% and 85% of the maximum HR, assessed by the graded exercise test (GXT). During physical exercise, BP, HR and symptoms of cardiovascular alterations were monitored. The speed and inclination were constantly adjusted to keep HR within the training range.

#### Measurements

All groups were assessed at baseline, end of interventions (3 months), and 6 months later (9 months). The assessment comprised physical, metabolic, behavioral and psychological aspects of the individuals studied.

#### Sociodemographic data

Data on personal identification, psychosocial and health aspects, such as diagnosis, medications in use and lifestyle (smoking habit, use of alcohol, physical activity), were collected in individual interviews by use of a structured questionnaire.

Alcohol use: male intake - up to 1 oz (30 ml) of ethanol/day; female intake - up to 0.5 oz of ethanol/day.^26^

Physical activity: exercise at least once a week as opposed to no exercise, the latter characterizing a sedentary lifestyle.

#### Clinical parameters

The anthropometric profile assessment included measuring WC, with a millimeter non-extensible long tape at the abdomen's maximum extension,^27^ body weight, and height, to calculate body mass index (BMI). Individuals were barefoot and lightly dressed having body weight measured, through the use of a properly calibrated 160-kg Cauduro^®^ scale. The Sunny^®^ vertical anthropometer was used for measuring height. Blood pressure values were assessed in three consecutive measurements, according to the American Hypertension Guidelines.^26^

#### Laboratory parameters

Blood samples were collected after fasting for the analysis of biochemical markers. Plasma and serum were separated and stored at -80°C for later analyses at HSL-PUCRS' laboratory. The tests analyzed were FG, total cholesterol, HDL-C and TGL, while low-density protein was determined indirectly.

#### Depression and anxiety

These variables were measured through the Adult Self Report (ASR),^28^ self-administered scale of 126 items that aims to identify the aspects of adults' adaptive functioning between the ages of 19 and 59 years, identifying behavioral and emotional problems and higher incidence of psychopathological disorders, such as anxiety and depression. Scores range from 0 to 100, with higher scores indicating a greater number of behavioral problems. Individuals with scores above 60 within the internalization scale, who demonstrate borderline and clinical status or under drug treatment, were classified as depressed or anxious.

#### Quality of life

This variable was assessed using the Medical Outcomes Study Short Form, General Health Survey (SF-36)^29^ that evaluates the QOL of individuals in relation to their disease. It consists of 36 questions, divided into the following 8 domains: physical functioning; limitations due to physical problem; bodily pain; general health perceptions; vitality; social functioning; role limitations due to emotional problems; and mental health. These domains were summarized into Physical and Mental Component Summary (PCS and MCS, respectively). The scores range from 0 to 100 for each domain, in which higher scores indicate better QOL.

### Statistical analysis

For α = 0.05, 90% power and estimating a difference between WC averages of 0.9 units of standard deviation, a sample number of 27 volunteers in each group was calculated. Considering maximum loss of 20%, the sample size became 34 per group.

Quantitative data were described as mean and standard deviation. Categorical variables were presented as counts and percentages. Comparisons of quantitative data used the one-way Anova for 3 groups and *t* test for 2 groups. For categorical data, we used the chi-square and Fisher's exact test, when necessary. To evaluate the outcomes, MetS components and QOL scores, considering adjustment for confounding factors, analysis of covariance and multiple linear regression were used. Additionally, analysis of covariance was used for comparisons at 3 and 9 months, adjusting for baseline measures and other confounding factors. The results were subjected to statistical analysis using the Statistical Package for Social Sciences (SPSS) program, version 21, with an alpha level of significance at 5%.

## Results

**Figure 1:**
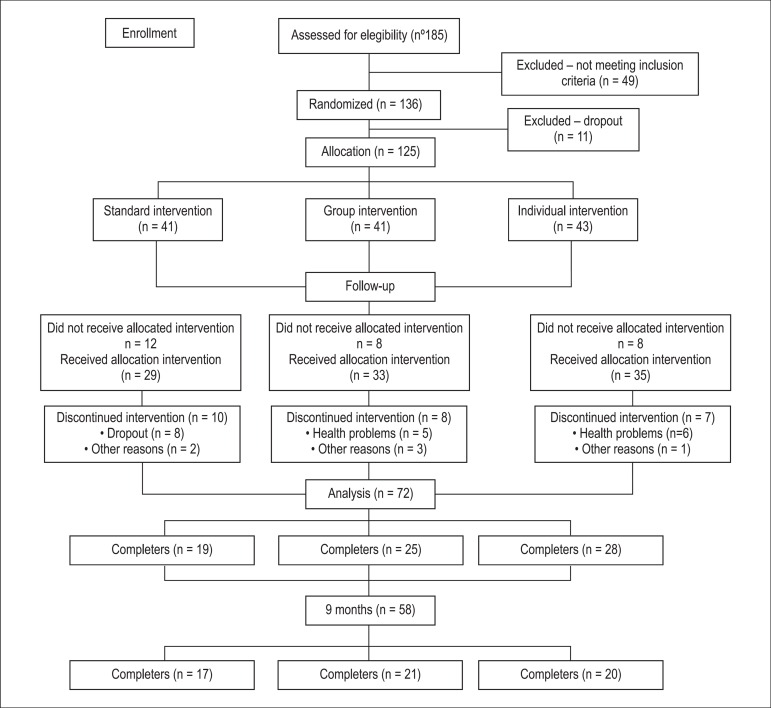
Flow chart of study participants.

This study included 72 individuals who concluded the intervention, divided into three groups: SI, 19; GI, 25; and II, 28 (Figure 1). Individuals who did not complete the trial and the ones that remained in the study showed similar characteristics regarding race, marital status and BMI. However, there were more women with lower levels of education (data not shown).

According to Table 1, most of the population studied was female, white and had high levels of education. Groups showed similar distributions in terms of general characteristics, as well as MetS and QOL components, without statistically significant differences at baseline.

**Table 1:** Baseline characteristics of the study participants

	SI	GI	II	p
	**n=19**	**n=25**	**n=28**	
Age, years	52.1±7.2	50.9±7.7	51.6±5.6	0.831 *
Female, n (%)	7 (36.8)	13 (52.0)	20 (71.4)	0.055 †
White, n (%)	17 (89.5)	23 (92.0)	24 (85.7)	0.763 †
**Marital status, n (%)**				0.768 †
With companion	12 (66.7)	15 (71.4)	17 (68.0)	
Single	3 (16.7)	5 (23.8)	6 (24.0)	
Widowed	3 (16.7)	1 (4.8)	3 (16.7)	
**Level of education, n (%)**				0.424 †
4 years of study	0 (0.0)	0 (0.0)	1 (3.6)	
5 to 8 years of study	0 (0.0)	0 (0.0)	1 (3.6)	
Over 9 years of study	19 (100.0)	25 (100.0)	26 (92.9)	
Sedentary lifestyle, n (%)	11 (57.9)	16 (64.0)	21 (75.0)	0.442 †
Smoking, n (%)	0 (0.0)	2 (8.0)	1 (3.6)	0.305 †
Use of alcohol, n (%)	0 (0.0)	2 (8.0)	2 (7.1)	0.280 †
BMI, kg/m^2^	33.5±4.1	35.1±3.6	33.7±3.2	0.283 *
**MetS Components**				
WC (cm)	112.6±8.3	112.9±10.0	110.7±7.2	0.605 *
SBP (mmHg)	132.6±10.3	131.8±15.2	135.5±13.5	0.577 *
DBP (mmHg)	90.6±10.3	89.7±12.7	89.2±11.6	0.922 *
TGL (mg/dL)	174.6±60.2	266.5±227.0	200.4±84.9	0.101 *
HDL-C (mg/dL)	46.4±8.9	47.7±11.3	48.2±14.1	0.872 *
**SF-36**				
Physical functioning	76.8±20.6	74.8±18.1	77.0±17.2	0.898 *
Role-physical	75.0±35.4	77.1±26.5	86.6±30.0	0.365 *
Bodily pain	62.8±21.9	63.6±21.5	70.9±22.8	0.369 *
General health	73.2±14.8	72.8±18.3	72.0±18.6	0.973 *
Vitality	58.9±22.9	61.0±22.7	58.9±22.0	0.933 *
Social functioning	82.4±23.5	78.3±23.8	80.8±16.3	0.810 *
Role emotional	80.7±25.6	72.0±39.3	70.2±38.8	0.600 *
Mental health	71.6±18.8	71.7±22.8	68.9±16.8	0.842 *
Physical component summary	46.8±8.5	47.2±6.8	49.9±5.5	0.227 *
Mental component summary	50.2±10.2	48.9±14.1	47.1±9.7	0.664 *

* ANOVA;

† Chi-square test; SI: standard intervention; GI: group intervention; II: individual intervention; BMI: body mass index; MetS: metabolic syndrome; WC: waist circumference; SBP: systolic blood pressure; DBP: diastolic blood pressure; TGL: triglycerides; HDL-C- High: density lipoprotein cholesterol.

**Figure 2:**
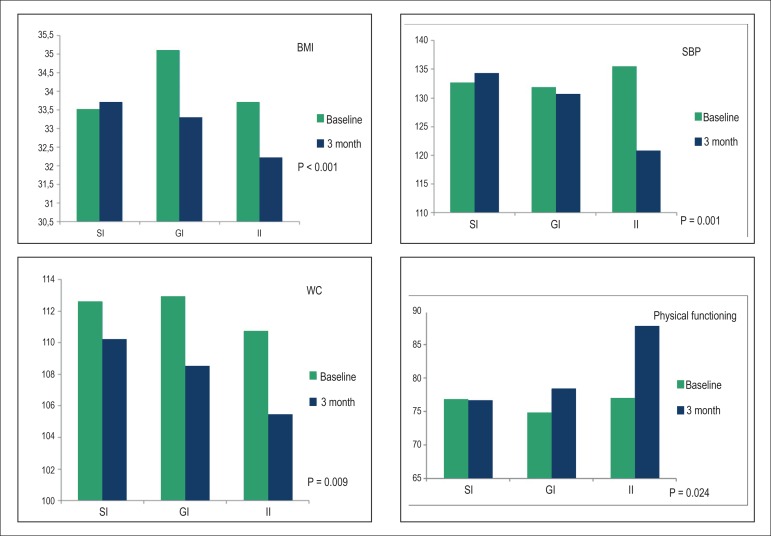
BMI, WC, SBP and Physical Functioning measures at baseline and 3 months. BMI: body mass index; WC: waist circumference; SBP: systolic blood pressure; SI: Standard Intervention; GI: Group Intervention; II: Individual intervention.

Table 2 presents results regarding MetS components in the three types of intervention. Although there was a reduction in TGL, FG and DBP, only BMI, WC and SBP showed significant reduction in their mean scores after 12 weeks. Compared to baseline, only II was associated with a significant reduction in SBP levels. On the other hand, regarding BMI and WC, both the GI and II showed a significant reduction in their mean scores, and GI was more effective in the reduction of BMI (Figure 2).

**Table 2:** Comparisons between the 3 groups at 3 and 9 months in metabolic parameters by ANCOVA

Variables	SI			GI			II		p	p *
	Month 3 (n=19)	Month 9 (n=17)		Month 3 (n=25)	Month 9 (n=21)		Month 3 (n=28)	Month 9 (n=20)		
**MetS Components**										
BMI (kg/m^2^)	33.7±0.3	33.2±0.4		33.3±0.3	33.5±0.4		32.2±0.2	32.4±0.4	<0.001	0.144
WC (cm)	110.2±1.2	108.0±1.3		108.5±1.0	108.0±1.1		105.4±1.0	106.4±1.1	0.009	0.522
SBP (mmHg)	134.3±2.8	132.9±3.9		130.6±2.5	128.8±3.3		120.6±2.3	124.6±3.6	0.001	0.330
DBP (mmHg)	86.4±2.5	85.6±2.2		84.6±2.2	82.8±1.8		80.9±2.0	80.7±1.9	0.199	0.263
TGL (mg/dL)	215.0±14.5	182.1±19.7		203.6±12.8	210.7±16.1		176.2±12.3	203.4±15.9	0.103	0.539
HDL-C (mg/dL)	43.6±1.4	45.2±1.7		48.0±1.2	47.4±1.4		46.9±1.2	46.3±1.4	0.060	0.616
FG (mg/dL)	111.0±4.3	112.3±5.3		107.7±3.6	106.9±4.4		99.5±3.7	105.6±4.3	0.108	0.600

p: statistical significance at 3 months; p *: statistical significance at 9 months; SI: standard intervention; GI: group intervention; II: individual intervention; MetS: metabolic syndrome; BMI: body mass index; WC: waist circumference; SBP: systolic blood pressure; DBP: diastolic blood pressure; TGL: triglycerides; HDL-C: highdensity lipoprotein cholesterol; FG: Fasting glucose.

Regarding QOL scores, almost all domains in all types of intervention showed an increase in their mean scores after 12 weeks. However, only physical functioning showed significant association (p=0.024), although general health had borderline significance. Compared to baseline, in almost all SF-36 domains, QOL improvement was higher in the II, although no statistically significant difference among the groups was found. Considering the PCS and MCS scores, no significant difference was found after the intervention. Similarly, there was no significant difference among the groups, despite the fact that II encouraged a larger increase in the MCS (Table 3).

**Table 3:** Comparisons between the 3 groups at 3 and 9 months in the QOL domains by ANCOVA

Variables	SI		GI		II		p	p *
	Month 3 (n=19)	Month 9 (n=17)	Month 3 (n=25)	Month 9 (n=21)	Month 3 (n=28)	Month 9 (n=20)		
**SF-36**								
PF	76.7±3.5	76.8±4.5	78.3±3.0	78.5±4.1	87.7±2.8	75.3±4.1	0.024	0.865
RP	83.6±5.3	86.5±9.0	92.1±4.7	73.7±8.1	88.4±4.4	82.7±8.2	0.488	0.543
BP	72.8±5.0	70.5±5.4	73.3±4.4	65.9±4.8	79.3±4.2	61.7±5.1	0.511	0.513
GH	79.6±2.8	79.5±3.9	78.0±2.5	76.2±3.5	85.8±2.3	78.8±3.6	0.057	0.799
VT	69.5±3.1	66.9±4.5	71.2±2.7	64.3±4.1	77.6±2.6	68.9±4.2	0.096	0.732
SF	84.2±4.2	78.9±5.3	87.2±3.6	78.5±4.8	92.7±3.5	81.2±5.1	0.272	0.922
RE	88.8±6.8	79.6±7.3	80.2±5.9	87.9±6.6	88.4±5.6	81.7±6.7	0.522	0.676
MH	77.8±3.0	72.9±3.9	76.1±2.6	77.3±3.5	82.7±2.5	75.1±3.6	0.163	0.708
PCS	49.2±1.5	50.3±2.3	50.6±1.3	46.7±2.0	51.8±1.3	47.2±2.2	0.444	0.477
MCS	53.2±1.8	49.8±2.2	52.2±1.6	52.5±2.0	55.2±1.5	52.4±2.1	0.377	0.606

p: statistical significance at 3 months; p *: statistical significance at 9 months; SI: standard intervention; GI: group intervention; II: individual intervention; SF-36: Medical Outcome Study Short Form General Health Survey; PF: physical functioning; RP: Rrle-physical; BP: bodily pain; GH: general health; VT: vitality; SF: social functioning; RE: role-emotional; MH: mental health; PCS: physical component summary; MCS: mental component summary.

Nevertheless, these results concerning the improvement of metabolic parameters, as well as QOL, were not kept 6 months after the end of intervention.

The prevalence of anxiety and depression was 41.7% and 22.2%, respectively. Regarding metabolic parameters, there was no significant association between MetS components and depression and anxiety. Concerning QOL, the mean scores for individuals with anxiety were lower in all SF-36 domains compared to those who did not have anxiety, although they were significant only in 5 domains (Table 4).

**Table 4:** Averages of the SF-36 scores of individuals with depression (DEP) and without depression (N-DEP) and with anxiety (ANX) and without anxiety (N-ANX)

Variables (n=72)	DEP	N-DEP	p	ANX	N-ANX	p
**SF-36**						
Physical functioning	73.1±5.3	77.0±2.3	0.502	75.0±3.2	77.0±2.9	0.643
Role-physical	54.7±8.9	87.7±3.3	0.002	76.7±5.2	82.9±5.0	0.388
Bodily pain	52.2±4.2	70.2±2.9	0.001	59.1±3.5	71.3±3.5	0.016
General health	65.7±4.8	74.6±2.2	0.107	70.9±2.8	73.8±2.9	0.471
Vitality	45.9±5.3	63.6±2.8	0.007	51.7±3.6	65.3±3.4	0.008
Social functioning	64.4±6.9	85.0±2.2	0.011	72.3±4.4	86.2±2.5	0.009
Role-emotional	45.8±10.0	81.5±4.1	0.004	61.1±7.5	82.5±4.4	0.018
Mental health	53.5±5.5	75.4±2.1	0.001	61.7±3.8	76.9±2.4	0.001

t test; SF-36- Medical Outcome Study Short Form General Health Survey.

Among the individuals who had depression, besides the lower QOL mean scores, all QOL domains, except for physical functioning and general health, showed significant difference when compared to those who did not have depression (Table 4). Regarding the influence of anxiety and depression in the intervention response, this study demonstrated that only depression had a negative significant effect on the scores of the SF-36 role emotional domain, although there was no statistical difference among the groups analyzed. MetS components, as well as the other QOL domains, showed no association with depression and anxiety.

## Discussion

This study tested 3 types of multidisciplinary intervention for lifestyle change in individuals with MetS, followed during 9 months, to determine its effects in the reduction of metabolic parameters and improvement of QOL. Results suggest that GI, as well as II were associated with significant BMI and WC reduction although only II had been significantly associated with lower SBP levels, which partially confirms the hypothesis previously established. It was surprising that GI reduced BMI levels more than II. According to a previous study,^30^ which demonstrated that standard advice is not sufficient to obtain changes in lifestyle and cardiovascular risk factors, SI, even showing a slight reduction in WC, did not have positive results in the improvement of the other metabolic parameters or statistically significant improvements in QOL, and II and GI showed a better response to intervention. Despite the fact that GI showed a smaller WC reduction than II, considering the fact that a 3-cm reduction already results in significant improvement of cardiometabolic risk factors,^31^ GI proved effective once it promoted a 4.4-cm reduction.

In accordance with previous reports,^7-9,15^ this study demonstrated that lifestyle intervention produced beneficial effects on metabolic parameters, especially on weight loss and WC, and the average of WC reduction in the II group was similar to the one found in individuals who underwent an intensive lifestyle intervention program.^8^ However, the present study, including all interventions, did not show statistically significant effects on FG, TGL,^32^ DBP and HDL-C.^8,32^ Although this study demonstrated improvement in QOL in both groups after intervention, in accordance with previous studies,^7-10,15-17,32^ only the physical functioning domain, also shown in other studies,^7,10^ showed a significant association. However, opposing data from most studies which demonstrated that QOL improvement is maintained after intervention for a period of 12,^9^ 24^17^ and up to 36 months,^7^ this study showed this effect only after the end of intervention.^32^ Due to the fact that there are no studies comparing the different approaches for individual and group lifestyle interventions in individuals with MetS, the finding that II showed higher effect on most QOL domains suggests that this result might be attributed to the intensity of intervention. This occurs because, according to the results of previous studies comparing types of intervention related to their intensity (moderate x intensive), the individuals who took part in more intensive programs showed significantly better results in weight reduction^33^ and in most QOL domains.^8^ Similarly, it is inconclusive whether this improvement in QOL might be related to weight loss, due to the relationship between BMI increase and QOL impairment,^34^ improvement in the physical condition^16^ or both.^10^

Another important contribution of this study is the fact that it demonstrated the influence of depression and anxiety in the reduction of scores in most QOL domains for individuals with MetS. Previous studies have already shown the association between MetS and depression and anxiety,^18-23^ but only a few analyzed its impact on QOL.^35^ Despite the fact that there was no significant influence of these variables in the response to intervention, deserves attention, as these clinical situations lead to QOL impairment, which justifies the importance of screening individuals with MetS for depression and anxiety.

This study provides preliminary data that a group intervention program can present results similar to individual intervention and, for this reason, might be an important prevention strategy, although its effects were not kept after the intervention. Therefore, it seems important to carry out a regular follow-up, as well as measures that encourage individuals to continue the lifestyle changes to maintain these effects. Moreover, group programs for lifestyle change seem to be an alternative intervention strategy that presents the best cost-benefit ratio in the management of metabolic parameters, as well as QOL of individuals who suffer from this important clinical condition nowadays.

A limiting factor in this study was the dropout rate, which hindered the use of the intention to treat analysis. Although this rate was similar between GI and II interventions, SI presented a high figure. A possible explanation for this can relate to the fact that the SI did not meet the individuals' expectations, since they were looking for a new type of intervention. Although the dropout occurred during follow-up, individuals who did not complete the study showed no significant differences when compared to individuals who remained in the study, which might minimize the effect of these losses. Another limiting factor concerns the relatively small intervention period of 12 weeks. Although this is the period normally used in other trials, metabolic parameters and QOL improvement results might have been kept if the intervention had lasted longer.

## Conclusion

Multidisciplinary intervention, especially in a group, might be an effective and economically feasible strategy to control the metabolic parameters of MetS and improvement of QOL compared to SI, even in a dose-effect relationship.
